# Wing-kinematics measurement and aerodynamics in a small insect in hovering flight

**DOI:** 10.1038/srep25706

**Published:** 2016-05-11

**Authors:** Xin Cheng, Mao Sun

**Affiliations:** 1 Institute of Fluid Mechanics, Beijing University of Aeronautics & Astronautics, Beijing 100191, China

## Abstract

Wing-motion of hovering small fly *Liriomyza sativae* was measured using high-speed video and flows of the wings calculated numerically. The fly used high wingbeat frequency (≈265 Hz) and large stroke amplitude (≈182°); therefore, even if its wing-length (*R*) was small (*R* ≈ 1.4 mm), the mean velocity of wing reached ≈1.5 m/s, the same as that of an average-size insect (*R* ≈ 3 mm). But the Reynolds number (*Re*) of wing was still low (≈40), owing to the small wing-size. In increasing the stroke amplitude, the outer parts of the wings had a “clap and fling” motion. The mean-lift coefficient was high, ≈1.85, several times larger than that of a cruising airplane. The partial “clap and fling” motion increased the lift by ≈7%, compared with the case of no aerodynamic interaction between the wings. The fly mainly used the delayed stall mechanism to generate the high-lift. The lift-to-drag ratio is only 0.7 (for larger insects, *Re* being about 100 or higher, the ratio is 1–1.2); that is, although the small fly can produce enough lift to support its weight, it needs to overcome a larger drag to do so.

To be airborn, an insect needs to generate a lift that is equal to its weight. An insect beats its wings to produce a velocity relative to the air (relative velocity), and hence the required aerodynamic force. Although the wingbeat frequency is high^1^, the relative velocity is low because of the small wing length. Consequently, the required lift coefficient is high, which is of the order of 2^2^,^3^,^4^. On the other hand, owing to the small size and velocity, the Reynolds number (*Re*) of an insect-wing is low, below 4000^4^,^5^,^6^,^7^,^8^. For a wing operating at steady-state condition, as the Reynolds number becomes lower, its ability to produce lift is greatly reduced^9^,^10^; for example, at *Re* = 10^4^ the maximum lift coefficient of a thin airfoil is about 0.8^9^, and at *Re* = 100, the maximum lift coefficient of a model fruitfly wing is about 0.6^10^. The high-lift coefficient of insects’ wings at low *Re* must be produced by unsteady flow effects^2^.

Much work has been done for revealing the unsteady high-lift mechanisms of insect wings (e.g. refs 5, 6, 7, 8 and 11, 12, 13, 14, 15, 16, 17, 18, 19, 20, 21). It was shown that the major unsteady mechanism that produced the high-lift coefficient at low *Re* was the delayed stall mechanism; how this mechanism works is explained as following. During the flapping motion, an insect-wing generally moves at high angle of attack, around 35°. For an airplane wing moving at constant speed, when its angle of attack is increased to such a large value, its flow on the upper-surface of the wing would separate and a vortex is formed near the leading edge. A large lift can be produced by the vortex; but in a short time the vortex detaches from the wing and the lift would be lost, and the wing is said to be stalled. For the insect wing, a leading-edge vortex (LEV) is also formed, but the vortex does not detach from the wing in an entire downstroke or upstroke. Thus large lift produced by the vortex can be maintained. Because the stall is delayed permanently, this mechanism is called delayed-stall mechanism. Since the LEV plays a very important role in the delayed-stall mechanism, much work has been conducted to study its fluid-dynamic properties, which have shown the detailed structure of the LEV and how it changes with *Re* (e.g. refs 22 and 23), the aspect ratio of wing (e.g. refs 24 and 25) and the radius of gyration^26^, and have partially explained why the LEV remains attached throughout the stroke (e.g. refs 27, 28, 29, 30). There are some other high-lift mechanisms^5^,^6^,^7^,^8^, e.g. the added-mass and rotational mechanisms, but they play a less important role than the delayed-stall mechanism.

Average insect wing length is about 3–4 mm^1^. But most of the previous studies were made on insects of relatively large size, for example, locusts^31^,^32^, hawkmoths^33^,^34^, dragonflies^35^,^36^, bumblebees^37^,^38^, droneflies^19^ and hoverflies^39^,^40^. For a very small insect, *Encarsia formosa*, Weis-Fogh^41^ found that at the dorsal stroke-reversal, its wings performed a special motion, “clap and fling”: near the end of the upstroke, the wings move dorsally toward each other and when their leading edges are near each other, they rotate about their leading edges (‘clap’); then, at the start of the subsequent downstroke, the wings open by rotating about their trailing edges (‘fling’). Some butterflies also use the fling motion during take-off (e.g. ref. 42). Flows and aerodynamic forces produced by the ‘clap and fling’ motion at the *Re* of the *Encarsia formosa* were studied in detail [e.g. refs 43, 44, 45, 46, 47, 48]. But since only one camera was used in Weis-Fogh’s study, detailed wing kinematics in a whole stroke cycle could not be measured. Fruit-flies (*Drosophila*) are so far the smallest insects whose detailed wing kinematics have been measured and aerodynamics studied (e.g. refs 10,17,21,49, 50, 51, 52, 53). Fruit-flies (*Drosophila virilis* and *D*. *melanogaster*) in these studies^10^,^17^,^21^,^49^,^50^,^51^,^52^,^53^ have a wing length on the order of 2.5–3 mm and they are of average-size insects. Some smaller fruit-flies (e.g. *D. nikananu*) were studied in ref. 52. Wing motion and aerodynamic force were measured and their relationship was analyzed. But because the flow-field was not measured, the unsteady mechanism was not explored; furthermore, the insects were tethered in the experiment, and the tethering could affect the wing kinematics^17^. We thus see that about half of the existing insects are smaller than fruit-flies *D. Virilis* and *D. melanogaster*, but their detailed wing kinematics are not known and their mechanics of flight has not been investigated. Given the large number of these small insects and their ecological and biological significance, it is of great interest to study their flight mechanics.

In the present work, we analyzed the detailed wing kinematics and aerodynamics of a small insect, vegetable leafminer (*Liriomyza sativae*), in hovering flight. Its wing length is about 1.4 mm. The reasons we chose the vegetable leafminers were that it is one of the smallest species in the order of *Diptera*^1^, and that they perform well hovering in the laboratory conditions. We first measured the time course of the wing motion, using three high-speed cameras and also measured the morphological data required for aerodynamic analysis. Then, with the measured wing kinematics, we computed the aerodynamic forces of the wings by the method of computational fluid dynamics. Analyzing the wing motion and aerodynamic forces could provide insights into how the forces are produced.

## Results and Discussion

Hovering in five vegetable leafminers (denoted as VL1, VL2, VL3, VL4 and VL5, respectively) was filmed. Samples of the original video sequences for a fly (VL1) are presented as supplementary material, Supplementary Movie 1. For each of the flights, films of four to six wingbeats were digitized.

### Morphological parameters

Morphological parameters of the insects are given in Table 1, including the wing length (*R*), mean-chord length (*c*), the area of one wing (*S*), the radius of gyration (*r*_2_), and the total mass (*m*). As seen in the table, the wing length (*R* ≈ 1.4 mm) is less than a half of that of a fruit-fly *D. virilis* (*R* ≈ 3 mm^21^) and less than 1/7 of that of drone-fly (*R* ≈ 11 mm^19^).

### Wing kinematics

**Figure 1:**
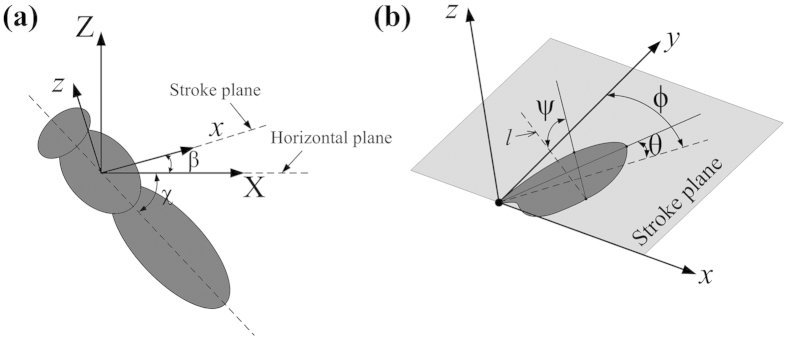
Reference frames and Euler angles of a flapping wing. *l*, a line that is perpendicular to the wing span and parallel to the stroke plane. *ϕ*, *θ* and *ψ*: positional angle, deviation angle and pitch angle of the wing, respectively.

We employed the method given by Ellington^37^ to describe the wing kinematics of the flies. In each wingbeat, *m* (about 24) pictures were taken. In a reference frame fixed on the body of the insect, *m* points on the curve traced by the wing-tip was recorded. The points traced by the tips of both the left and right wings were projected onto the plane of symmetry of the insect. A linear regression line of the projections was then determined. The plane which is parallel to the above line and passes the wing base is defined as stroke plane. Let (*X*, *Y*, *Z*) be a reference frame with origin at the wing base, the *X* axis being horizontal and pointing backward, the *Z* axis being vertical and pointing upward and the *Y* axis pointing to the right of the insect (Fig. 1). Let (*x*, *y*, *z*) be another reference frame with origin at the wing base, with the *x*-*y* plane coinciding with the stroke plane, the *z* axis normal to the stroke plane, and the *y* axis pointing to the right of the insect (Fig. 1). The angle between the stroke plane and the horizontal, *β*, is referred to as stroke-plane angle. The angles determining the wing orientation relative to the stroke plane are defined as follows. A line is drawn passing the wing-base and wing-tip (Fig. 1). The orientation of the wing is determined by the three Euler angles: positional angle (*ϕ*), stroke deviation angle (*θ*) and pitch angle (*ψ*) (Fig. 1), where *ϕ* is defined as the angle between the projection of the line joining the wing-base and wing tip onto the stroke plane and the *y*-axis, *θ* is defined as the angle between the line joining the wing-base and wing-tip and its projection onto the stroke plane, and *ψ* is defined as the angle between the local wing chord and *l*, a line that is perpendicular to the wing span and parallel to the stroke plane. The pitch angle *ψ* is related to the angle of attack of the wing (*α*) as following: *α* = *ψ* in the upstroke and *α* = 180°−*ψ* in the downstroke.

The measured wingbeat frequency *n* and stroke-plane angle *β* for the five flies are given in Table 2. The body angle (*χ*), angle between the long-axis of body of the insect and the horizontal was also obtained; *β* + *χ* is the angle between the stroke plane and the long-axis of the insect body (Fig. 1), which is also given in Table 2.

**Figure 2:**
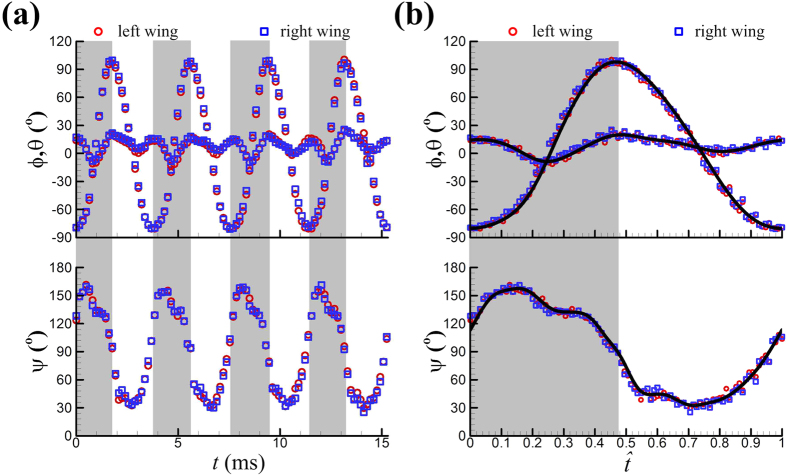
Wing kinematics of vegetable leafminer VL1. (**a**) Instantaneous wing kinematics of VL1. *ϕ*, positional angle; *θ*, stroke deviation angle; *ψ*, pitch angle. Grey bars represent the upstroke (*t* represents time). (**b**) Filtered values of *ϕ*, *θ* and *ψ* of VL1 as functions of time (the curves), compared with the original data (data of four cycles are superposed).

**Figure 3:**
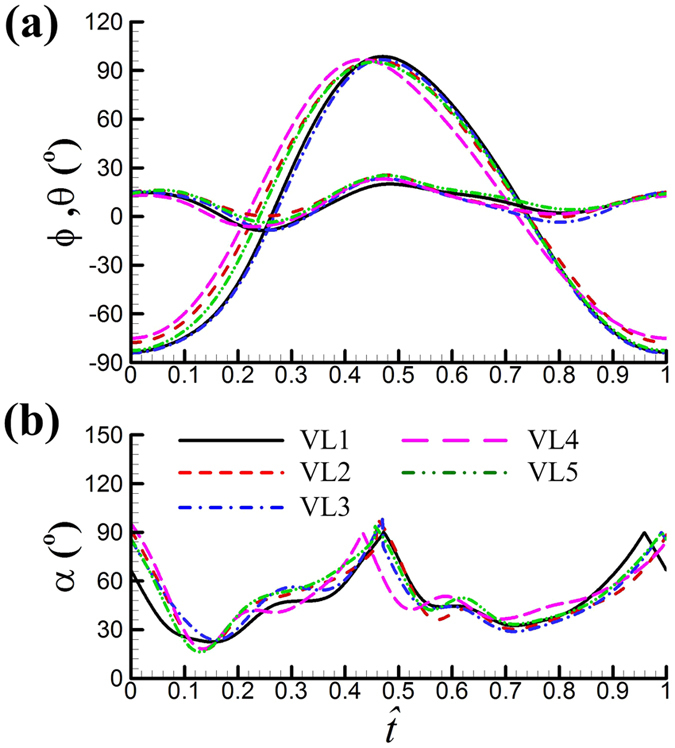
Filtered values of *ϕ*, *θ* and *α* in one cycle for each of the five vegetable leafminers.

The measured time courses of the Euler angles, *ϕ*, *θ* and *ψ*, of the wings of one of the insects, VL1, are shown in Fig. 2a. To eliminate the measurement noise, the *ϕ*, *θ* and *ψ* data in Fig. 2a were treated as following. First data at each time step are averaged between the left and right wings; then they were phase-averaged over the four wingbeat cycles; and finally the phase-averaged data were filtered using a fifth-order low-pass Butterworth filter (the cut-off frequency was 625 Hz, 1250 Hz, and 1875 Hz for *ϕ*, *θ*, and *ψ*, respectively). For a clear description of the data, we define a non-dimensional time (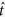): 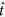 = 0 at the start of an upstroke and 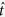 = 1 at the end of the subsequent downstroke. The filtered values of *ϕ*, *θ* and *ψ* as functions of 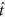 are given in Fig. 2b (the curves). The original data of four cycles are superposed and also plotted in Fig. 2b. It is seen that the curves well represent the original data. The *ϕ*, *θ* and *ψ* data of the other four flies, VL2, VL3, VL4 and VL5 were treated in the same way. The results for each of the flies are given in Fig. 3 (in the figure, instead of *ψ*, *α* is given).

In an upstroke, *ϕ* changes from its minimum value, *ϕ*_min_, to its maximum value, *ϕ*_max_ and in a downstroke, it is the other way around; the difference between *ϕ*_max_ and *ϕ*_min_ is the stroke amplitude, Φ. The value of *ϕ*_max_ determines how close to each other the left and right wings will be at the dorsal stroke-reversal, and the value of Φ (together with the wingbeat frequency *n*) determines the mean velocity of the wing. For convenience, the values of *ϕ*_max_, *ϕ*_min_ and Φ (obtained from Fig. 3) for each of the flies are also listed in Table 2.

From the data in Fig. 3 and Table 2, the following observations can be made. The flies use relatively high wingbeat frequency and large stroke amplitude: *n* ≈ 265 Hz and Φ ≈ 180° (for fruit-fly *D. virilis*, *n* ≈ 160 Hz and Φ ≈ 160° 21). The relative velocity of the wing is proportional to Φ, *n* and *R*. The Reynolds number of the wing is proportional to the relative velocity and the wing chord length, and the aerodynamic force on the wing is proportional to square of the relative velocity and the wing area. Let *U* be the mean velocity at the radius of gyration of the wing, *Re* be the Reynolds number of the wing and *F* be aerodynamic force on the wing, we have:


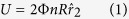



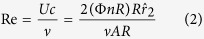



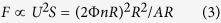


where 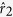 =  *r*_2_/*R*, *S* is the area of a wing (*S* = *Rc*), *AR* is the aspect ratio of the wing (*AR* = *R*/*c*) and *ν* is the kinematic viscosity of the air (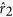 and *AR* do not vary greatly among different insects; 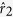 is about 0.6 and *AR* about 3 for many insects). For a vegetable leafminer, *R* is relatively small (Table 1) and using larger Φ and *n* could keep the relative velocity from being too low, so that the *Re* and the aerodynamic force from being too small. As aforementioned, fruit-flies *D. Virilis* and *D. melanogaster* were so far the smallest insects whose detailed wing kinematics was measured and aerodynamics studied. Let us make some comparisons between the present vegetable leafminers and the fruit-flies. For a fruit-fly (*D. virilis*) with *R* ≈ 2.9 mm, *n* ≈ 160 Hz and Φ ≈ 160° 21, *U* is about 1.53 ms^−1^, *Re* is about 100 and the lift produced is approximately equal to the weight^21^. For the vegetable leafminers, for example, VL1, *R* (1.4 mm) is less than half of that of the fruit-flies (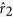 and *AR* are approximately the same as those of the fruit-flies). If the same Φ and *n* as those of the fruit-flies were used, *U* would be 0.74 ms^−1^, *Re* would be 21 and lift produced might be too small, because lift is proportional to the square of *U*. But with the actual Φ and *n*, the value of *U* is 1.49 ms^−1^ and *Re* is about 40. We see that by using relatively larger *n* and Φ, a vegetable leafminer makes its *U* (1.49 m/s) about the same as that of a fruit-fly (1.53 ms^−1^). With such a *U*, larger lift could be produced. Its Reynolds number (*Re* = 40), although increased by using the relatively large *n* and Φ, is still low, less than half of that of a fruit-fly and much less than other larger insects. In the next section, we will investigate whether or not the vegetable leafminers, whose *Re* is only about 40, use the same high-lift mechanisms to produce the required lift as those of the larger insects, whose *Re* is 100 and above.

**Figure 4:**
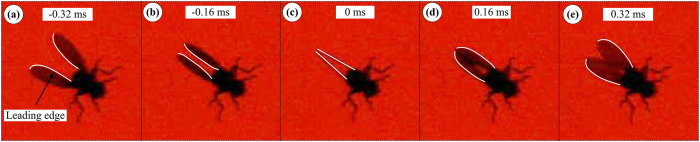
Successive snapshots showing the partial clap and fling of VL1. The images were recorded at 6250 frames per second.

**Figure 5:**
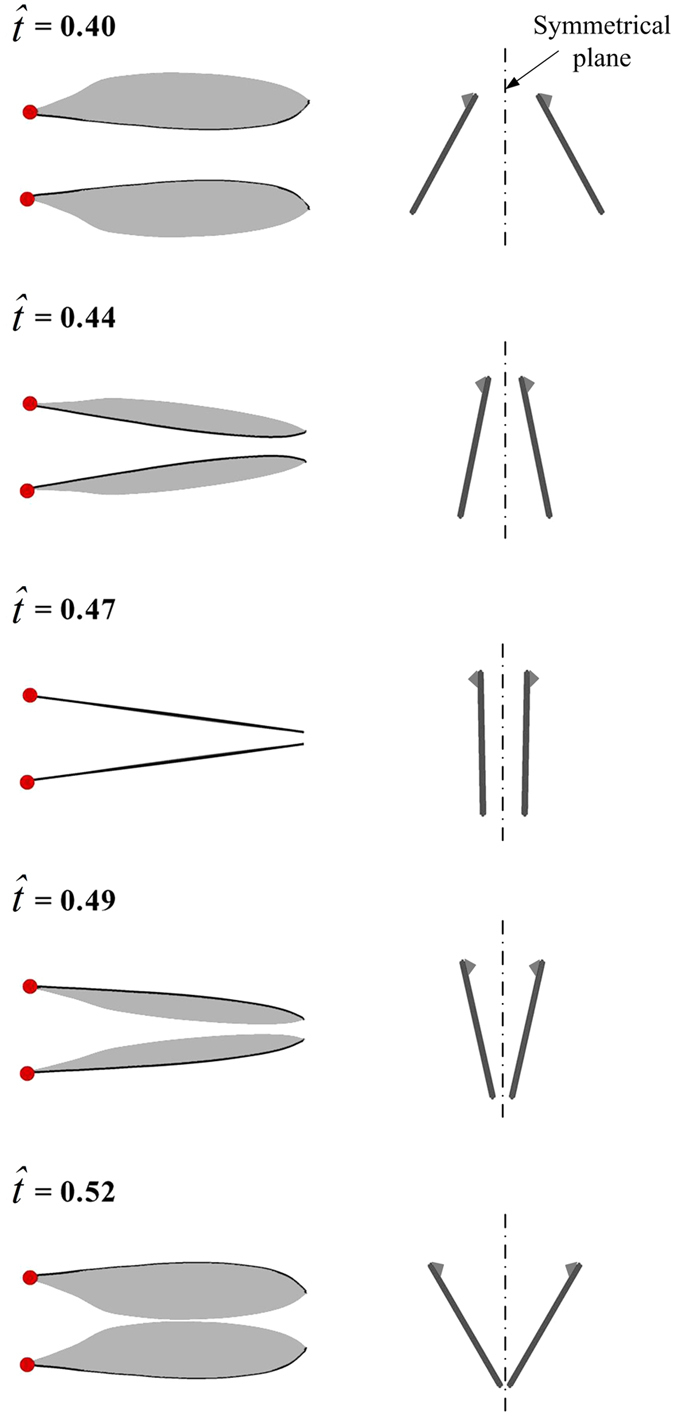
Wing motion of VL1 at the dorsal stroke-reversal.

Another observation is that in order to have a large Φ, the flies flap their wings to the extremes: *ϕ*_min_ is close to −90° and *ϕ*_max_ is more than 95°. When *ϕ*_max_ is larger than 90°, the outer parts of the wings would be in close proximity to each other at the dorsal stroke-reversal; i.e. the insect has a partial ‘clap and fling’ motion. Snapshots showing the partial clap and fling are given in Fig. 4. Using the wing motion data in Fig. 3, we plot the wing motion around the dorsal stroke-reversal in Fig. 5. It is seen that near the end of the upstroke (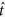 ≈ 0.4–0.47), the outer parts of the wings (approximately 0.75 *R* to 1 *R* from the wing root) have a motion similar to the clap motion, and in the beginning of the subsequent downstroke (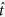 ≈ 0.47–0.52), the outer parts of the wings have a motion similar to the fling motion. How this partial clap and fling motion will affect aerodynamic forces will be examined in next section.

The flies were only in approximate hovering: they move at very small velocity (also measured). The non-dimensional velocity of the body motion is referred to as advance ratio *J* (defined as the velocity of motion divided by the mean wing-tip speed 2Φ*nR*). The values of *J* are also included in Table 2. Ellington proposed 0.1 as the limit to *J* for hovering flight^37^. As seen in Table 2, *J* of all the flies is less than that imposed by this limit, except that of VL4 (*J* of VL4, 0.124, is only a little larger than the limit).

The take-off flight of thrips was recently filmed using a high-speed camera^54^; flapping frequency (≈254 Hz), stroke amplitude (≈120°) and wing length (≈0.65 mm) were obtained from the film. Thus the wing-tip speed (2Φ*nR*) and *Re* could be calculated. It is of interest to compare these parameters between thrips and the flies. For the thrips, the mean wing-tip speed is about 0.68 m/s and the *Re* about 15; for the flies, the corresponding numbers are 2.5 m/s and 40. We see that the thrips, which have bristled wings, fly at much smaller wing-tip speed and *R*e than the flies.

### Computed flows and aerodynamic forces

Using the measured wing-motion data and the computational method described in the Materials and methods section and electronic supplementary material, flows around and aerodynamic forces acting on the wings of each of the flies were computed. Lift (*L*) is defined as the component of aerodynamic force of the wing that is perpendicular to the stroke plane and drag (*D*) as the component that is in the stroke plane and perpendicular to the wing span; vertical force (*V*) is the *Z* component of aerodynamic force. The coefficients of these forces are defined as: *C*_*L*_ = *L*/0.5 *ρU*^2^*S*, *C*_*D*_ = *D*/0.5 *ρU*^2^*S* and *C*_*V*_ = *V*/0.5 *ρU*^2^*S*, where *ρ* is the fluid density.

**Figure 6:**
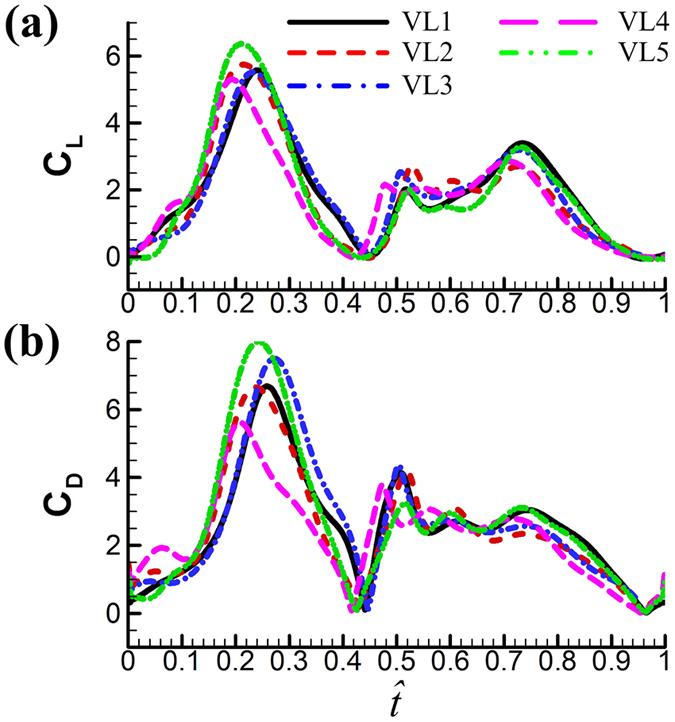
Time courses of the computed *C*_*L*_ and *C*_*D*_ in one cycle for the five vegetable leafminers.

**Figure 7:**
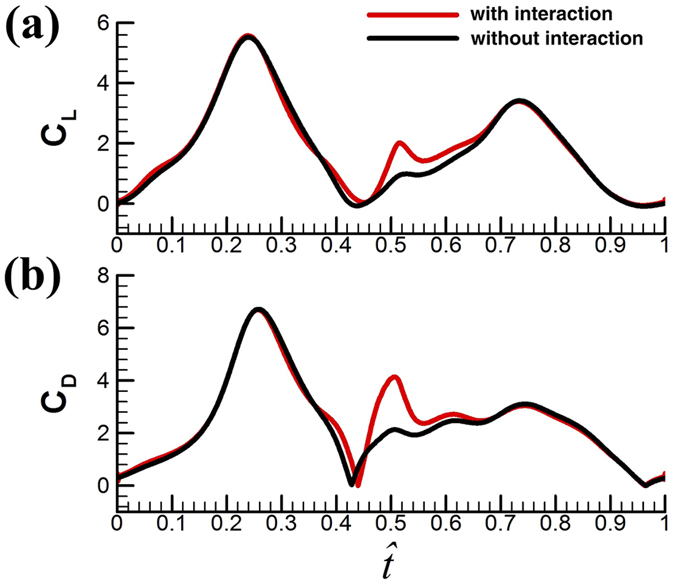
The time history of *C*_*L*_ and *C*_*D*_ for VL1 with and without aerodynamic interactions.

1. *Aerodynamic forces*. Figure 6 gives the time courses of the lift and drag coefficients in one cycle for the five flies. The time variations in the lift and drag coefficients are similar among the flies. This is because they have similar pattern of wing-motion (Fig. 3). Resolving *C*_*L*_ and *C*_*D*_ into the vertical direction gives the vertical force coefficient (*C*_*V*_). The mean vertical force coefficient (averaging *C*_*V*_ in one wingbeat cycle) is denoted by 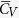. For VL1 to VL5, 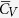 is 1.89, 1.92, 1.99, 1.71 and 1.78, respectively. Let the non-dimensional weight of a fly be denoted by *C*_*W*_, defined as *C*_*W*_ = weight/0.5 *ρU*^2^(2*S*). Values of *C*_*W*_ for the five flies are 1.71, 2.14, 1.68, 1.86 and 1.25, respectively. Comparing 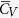 with *C*_*W*_ shows that for four of the five flies, 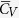 is different from *C*_*W*_ by about 10% (for the other one, VL3, the difference is about 20%). In general, the time-averaged vertical force is approximately equal to that needed to support the weight of the fly. The mean lift coefficient (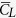) for the five flies are 1.84, 1.82, 1.93, 1.74, and 1.89, respectively. It is seen that 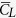 is not very different from 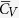 (because *β* is small) and that 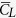 (and 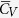) is on the order of 2, much larger than that of a cruising airplane. The mean drag coefficient (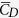) for the five flies are 2.56, 2.45, 2.60, 2.31, and 2.68, respectively. It is should be noted that 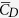 is about 40% larger than 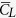 and the lift-to-drag ratio (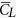/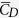) is only about 0.7. For larger insects in the previous studies (*Re* being about 100 or higher), 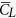/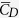 was 1–1.2; for example, for fruit-flies (*Re* ≈ 100), 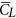/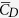 ≈ 1 21, and for droneflies (*Re* ≈ 780), 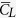/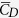 ≈ 1.2 19. We thus see that although the small flies in the present study can produce enough lift to support their weight, they need to overcome a larger drag to do so. Note that for the flies, the stroke amplitude has reached its maximum value (≈185°), and *α* is not small, around 40°. Could the flies increase the force coefficients by further increasing *α*? To see this, we have done some additional computations in which *α* of VL1 is increased (see Supplementary S4). The results show the following. Using the actual *α* (around 40°), 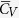 and 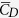 are 1.89 and 2.56, respectively. When *α* is increased by 11°, 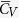 and 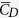 become 2.05 and 3.29, respectively (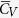
is increased by 8.5% and 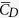 by 28.6%). When *α* is further increased, 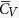 decreases and 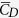 continue to increase. This shows that the fly can still increase its vertical force a little by increasing *α*, but to do so a much large drag must be overcome (i.e. at much larger energy expenditure).
2. *Effect of the ‘clap and fling*’. As discussed in the Wing kinematics section, the small flies have a partial ‘clap and fling’ at the dorsal stroke-reversal. In order to investigate the interference effect between the left and right wings in the partial ‘clap and fling’ motion, we made an additional calculation: computing the flow around a single wing performing the same flapping motion. This calculation represents the case of no aerodynamic interaction between the two wings. Figure 7 show the time history of *C*_*L*_ and *C*_*D*_ for VL1 with and without aerodynamic interactions. It is seen that during the ‘clap and fling’ motion (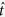 ≈ 0.44–0.54), the instantaneous *C*_*L*_ and *C*_*D*_ are increases by the interaction effect: in the period of 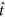 ≈ 0.48–0.52, the values of *C*_*L*_ and *C*_*D*_ are approximately doubled. However, because this period is rather short, the interaction could not increase the mean aerodynamic forces greatly: 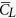 and 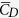 are increased by about 7% by the interaction effect. Similar results are obtained for the other four flies. We see that the partial ‘clap and fling’ motion only increases the aerodynamic force by a small amount. Lehmann *et al*.^47^ studied the partial clap and fling similar to that in the present work, employing robotic fruit-fly wings (*Re* ≈ 100). Their measurements showed that the lift could be augmented by about 9%, similar to our results. Although the partial clap and fling in the present and Lehmann *et al*.’s studies does not augment the lift greatly, the full clap and fling (the wings fully touch) does, as have been shown by many studies^45^,^46^,^47^,^48^,^55^,^56^. These studies showed that at *Re* about 15 (*R*e for the tiny insect *Encarsia formosa*), lift could be augmented by more than 30%^45^,^46^. The augmentation is very large and without it the insect may not be able to fly. It may be suggested that for tiny insect with membranous wings, when *Re* is at the order of 10, full clap and fling is required.
3. *How the aerodynamic forces are produced*. As seen above, the mean lift coefficient of the small flies is large, about 1.85. The previous section showed that the ‘clap and fling’ mechanism played only a minor role in the high lift generation. In this section, we investigate how the large aerodynamic forces are generated. Because the wing motion (Fig. 3) and the time histories of the aerodynamic forces (Fig. 6) are similar among the five flies, only the results of VL1 are analyzed.

As seen from the time histories of *C*_*L*_ and *C*_*D*_ in Fig. 7, the major part of the mean lift come from the mid-portions of the up- and downstrokes (see the large *C*_*L*_ peaks at 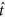 ≈ 0.1–0.4 and 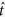 ≈ 0.58–0.9 in Fig. 7a). Using the data in Fig. 7a, it is estimated that about 86% of the mean lift is contributed by the mid-portions of the up- and downstrokes.

**Figure 8:**
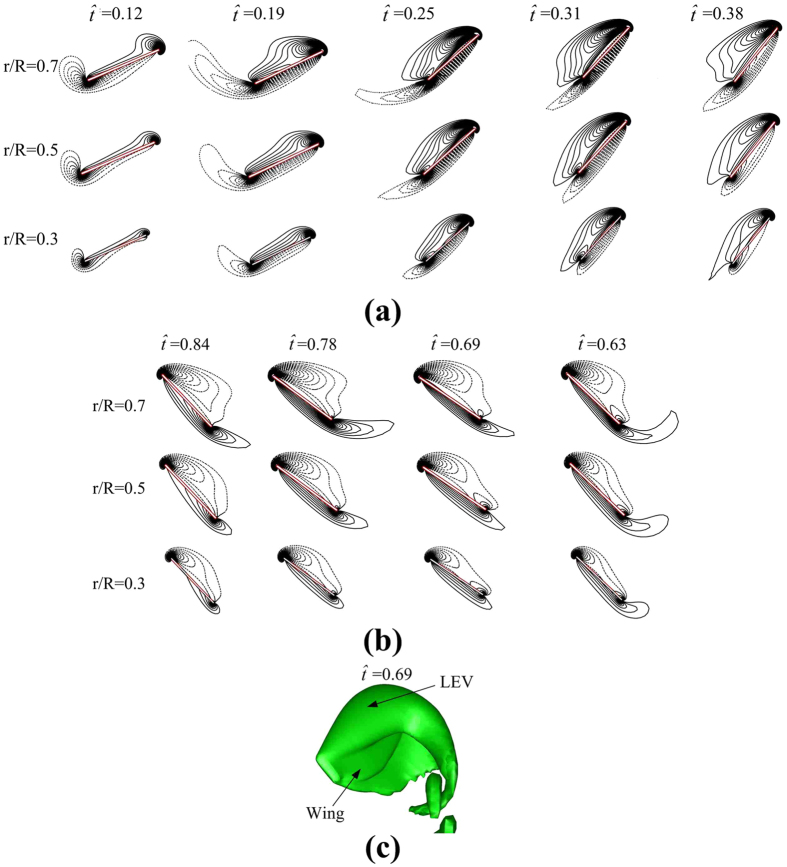
Contours plots of non-dimensional spanwise component of vorticity at different wing length and various times in the period of 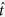 = 0.12–0.38 (**a**) and 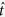 = 0.63–0.84 (**b**) (the magnitude of the vorticity at the outer contour is 2 and the contour interval is 1). (**c**) Iso-vorticity surface plot (top view) at an instant in the downstroke (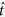 = 0.69), showing the three-dimensional structure of the LEV.

In the mid-portions of the up- and downstrokes (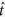 ≈ 0.1–0.4 and 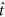 ≈ 0.58–0.9), the speed of the wing is approximately constant (see Fig. 3a: the slope of *ϕ*, i.e. d*ϕ*/d*t*, which represents the speed of the wing, has small variation at 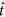 ≈ 0.1–0.4 and 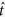 ≈ 0.6–0.9), and the angle of attack is large, about 40° (Fig. 3b). Let us examine flow-field data in these periods. Figure 8a shows the contour plots of the spanwise component of vorticity at three spanwise positions, at various times in the mid-portion of the upstroke (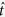 ≈ 0.1–0.4); Fig. 8b shows the corresponding results for the downstroke (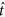 ≈ 0.58–0.9) (in Fig. 8a,b, solid and broken lines indicate counter clockwise and clockwise vorticity, respectively; the magnitude of the vorticity at the outer contour is 2 and the contour interval is 1). We see that as the wing moves with an approximately constant velocity at high angle of attack, there exists a leading-edge vortex (LEV) on the wing, and the LEV does not shed in the mid-portions of the up- and downstrokes (Fig. 8a,b) (a three-dimensional graph of the LEV at one time instant in the downstroke is shown in Fig. 8c). This shows that the large *C*_*L*_ and *C*_*D*_ in the mid-portions of the up- and downstrokes are due to the delayed-stall mechanism^11^,^12^. The above discussion show that the flies, although having a *Re* as low as about 40, still mainly use the delayed-stall mechanism to generate the required lift, as most other large insects do.

In the above discussions, the role played by the elevation angle (*θ*) is not mentioned. From Fig. 3a, it is seen that *θ* is relatively large, the peak-to-peak value is about 30° (for many insects, e.g. droneflies^19^,^37^ and hoverflies^37^,^40^, this value is about 10°). It is also seen that in a half-stroke (an upstroke or downstroke), *θ* is large at the beginning of the half-stroke; it decreases to a minimum value in the mid of the half-stroke and then increases. This results in a U-shape wing-tip path. That is, owing to the *θ* variation, the translating wing has a downward motion in the first part of a half-stroke and an upward motion in the next part of the half-stroke. In order to see the effect of *θ*, we made some additional computations, and the results and detailed analysis are given in Supplementary S5. The main results are as following. In the first part of a half-stroke, *C*_*L*_ and *C*_*D*_ are increased by the elevation angle (because the downward motion of the translating wing increases its effective angle of attack), while in the next part of the half-stroke, *C*_*L*_ and *C*_*D*_ are decreased by the elevation angle (because the upward motion of the wing reduces the effective angle of attack). Although the elevation angle produces the above effects, the general time-variations of the force coefficients are qualitatively similar between the case with actual *θ* and the case with zero *θ*.

## Conclusion

1. In hovering, the small fly vegetable leafminer uses relatively high wingbeat frequency (≈265 Hz) and large stroke amplitude (≈182°). Therefore, even if its wing-length is small (1.4 mm), the mean velocity at the radius of gyration of the wing reaches about 1.5 m/s, about the same as that of a fruit-fly (an average-size insect, *R* ≈ 3 mm); but *Re* of its wing is still low (≈40), owing to the small wing size (*Re* for average- and large-size insects is above 100).
2. In order to have a large Φ, the fly flaps their wings to the extremes: the maximum positional angle is more than 95°. As a result, the wings have a partial ‘clap and fling’ motion.
3. The computed mean lift is approximately equal to the weight, and the mean lift coefficient is high, about 1.85. The ‘clap and fling’ mechanism increases the lift only by about 7%. The small fly mainly uses the delayed stall mechanism to generate the required high lift coefficient, as most other larger insects do.
4. The lift-to-drag ratio of the small fly is only about 0.7 (for larger insects, *Re* being about 100 or higher, the lift-to-drag ratio is 1–1.2). That is, although the small fly can produce enough lift to support its weight, it needs to overcome a larger drag to do so.

## Materials and Methods

### Insects

Vegetable leafminers (*Liriomyza sativae* Blanchard) were obtained from the Laboratory of Biological Invasion of Institute of Plant Protection, Chinese Academy of Agricultural Sciences, which were descendents of wild-caught vegetable leafminers.

### Measurement of wing kinematics and morphological parameters

**Figure 9:**
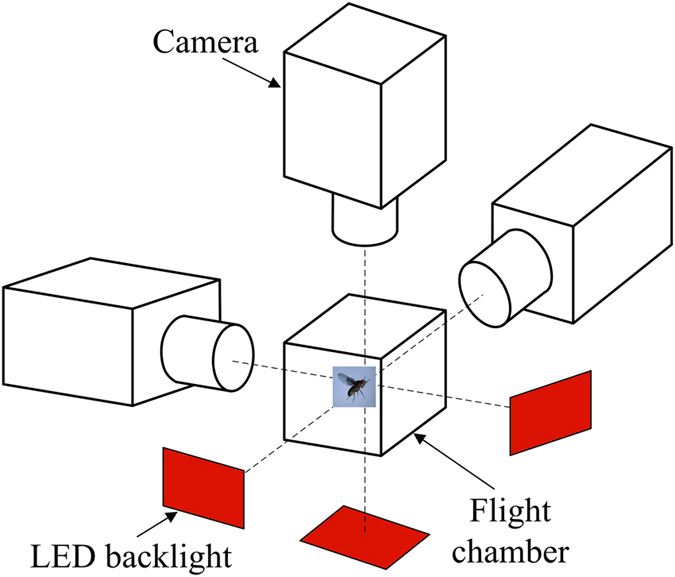
A sketch showing the flight chamber and cameras.

Hovering flight of vegetable leafminers was filmed in an enclosed flight chamber (Fig. 9) using three orthogonally aligned synchronized high-speed cameras (6,250 frames per second, shutter speed 20μs, resolution 464 × 272 pixels) mounted on an optical table. The flight chamber was built from plexiglass and had a size of 6 × 6 × 6 cm^3^, which was much smaller than that used for fruit-flies and drone-flies in our previous studies^19^,^21^,^40^. In the experiment, the flight chamber was in room light. Each camera view was backlit using a 50 W integrated red light emitting diode (LED) (luminous flux, 4000 lm; wavelength, 632 nm). Integrated LEDs were used as the light source because they produced much less heat than cine lights at the elevated light levels required for high-speed filming. The light was made uniform by two lenses. The intersecting field of views of the three cameras, a zone of approximately 0.75 × 0.75 × 0.75 cm^3^, was in the middle of the flight chamber; the size of this zone was also much smaller than that in the previous studies on fruit-flies and drone-flies^19^,^21^,^40^. Room temperature at the time of filming was 22 to 24 °C.

The method we employed to extract the three-dimensional wing (and body) kinematics from the filmed data was same as that used in several previous works of our group^19^,^21^,^40^. Mou *et al*.^40^ analyzed the errors of the method. In Mou *et al*.’s study, the camera speed was 5,000 frames per second, the resolution was 512 × 320 pixels, and the intersecting field of views of the three cameras was a zone of approximately 5 × 5 × 5 cm^3^; while in the present study, as mentioned above, the camera speed was 6250 frames per second, the resolution was 464 × 272 pixels, and the intersecting field of views of the three cameras was a zone of approximately 0.75 × 0.75 × 0.75 cm^3^. Because these differences, we needed to re-conduct error analysis of the method, which was described in the Supplementary Material. The analysis showed that errors in the positional angle were within 3° and errors in pitch angle and elevation angle were within 4° (see Supplementary S1).

**Figure 10:**
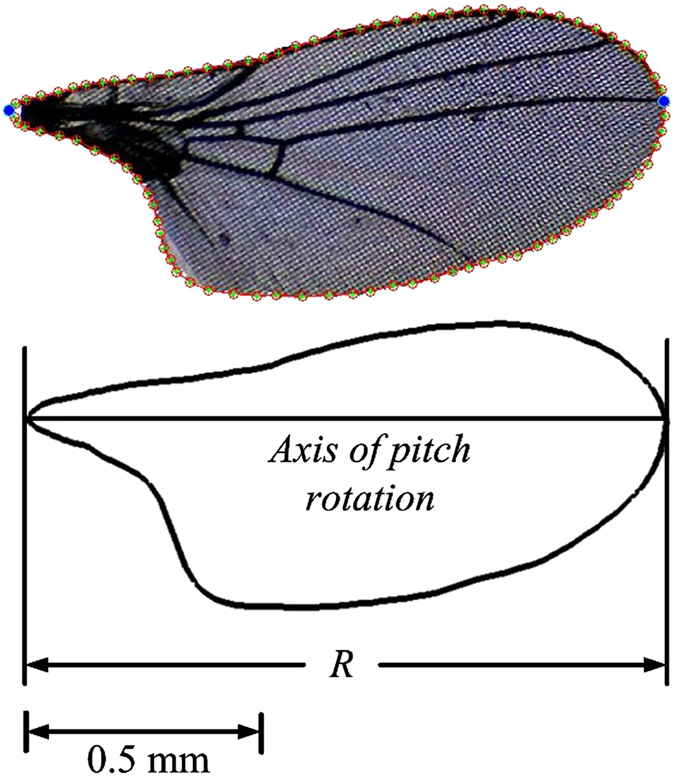
Wing model. *R*, wing length.

The morphological data required for aerodynamic analysis are: the wing shape, the wing length, the area of one wing, the radius of the second moment of wing area, or radius of gyration, and the total mass of the insect. The insects were killed with ethyl acetate vapor after filming. The total mass was measured to an accuracy ±0.01 mg. Then a wing was cut from the body. Using a microscope equipped with an electronic eyepiece (display resolution: 1280 × 1024), the shape of the wing under the microscope was captured from computer screen. A sample of the captured wing shape is shown in Fig. 10. Based on the picture, the wing length and local wing chord length were measured to an accuracy greater than ±1%. Wing area, mean chord length (*c*) and radius of gyration were computed using the measured wing length and local wing chord length.

### Computation of the flows and aerodynamic force

The flows around the insect were computed by numerically solving the Navier–Stokes equations. Using *c*, *U* and *c/U* as reference length, velocity and time, respectively, the dimensionless form of the Navier–Stokes equations is:


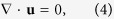



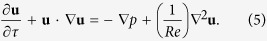


where **u** is the non-dimensional fluid velocity, *p* the non-dimensional fluid pressure, *τ* the non-dimensional time, and *Re* the Reynolds number. The aerodynamic force acting on the insect was obtained from the flow solution.

In principle, we should compute the flows around the wings and the body. But at hovering, because the velocity of the body was zero, the aerodynamic force of the body was negligibly small compared to that of the wings. Therefore, in the present computational model, the body was neglected. The wings are treated as rigid flat plates. For the small flies, the wing bending is rather small; this can be seen from the snapshots at the end of a downstroke or upstroke (see Supplementary Figs S3g,o).

In solving the Navier–Stokes equations, moving overset grids were used because the left and right wings were very close to each other at the dorsal stroke-reversal and there could be strong aerodynamic interaction between the wings. The numerical method was the same as that used in several previous studies of our group^13^,^36^,^45^. A description of it is given in Supplementary S3; the computational grids and the grid-resolution and time-step tests are also discussed there.

## Additional Information

**How to cite this article**: Cheng, X. and Sun, M. Wing-kinematics measurement and aerodynamics in a small insect in hovering flight. *Sci. Rep*. **6**, 25706; doi: 10.1038/srep25706 (2016).

## Supplementary Material

Supplementary Information

Supplementary Movie S1

## Figures and Tables

**Table 1:** Morphological parameters of the vegetable leafminers: total mass (*m*), wing length (*R*), mean chord length of wing (*c*), area of one wing (*S*) and radius of the second moment of wing area (*r*_2_).

ID	*m*(mg)	*R*(mm)	*c*(mm)	*S*(mm^2^)	*r*_2_/*R*
VL1	0.25	1.44	0.42	0.61	0.59
VL2	0.32	1.46	0.43	0.63	0.59
VL3	0.29	1.49	0.45	0.67	0.59
VL4	0.31	1.45	0.42	0.60	0.59
VL5	0.17	1.31	0.40	0.52	0.59

**Table 2:** Kinematic parameters of the vegetable leafminers: wingbeat frequency (*n*), stroke plane angle (*β*), body angle (*χ*), maximum value of positional angle (*ϕ*_max_), minimum value of positional angle (*ϕ*_min_), stroke amplitude (*Φ*), advance ratio (*J*) and Reynolds number (*Re*).

ID	*n*(Hz)	*β* (deg.)	*β *+* χ*(deg.)	*ϕ*_max_ (deg.)	*ϕ*_min_ (deg.)	Ф (deg.)	*J*	*Re*
VL1	260.8	−1.7	54.3	99.1	−83.3	182.4	0.042	39.7
VL2	270.4	−19.9	55.1	96.4	−77.7	174.1	0.096	39.1
VL3	259.3	−9.3	51.7	97.6	−84.1	181.7	0.090	42.9
VL4	288.4	−19.8	52.2	97.3	−75.1	172.4	0.124	41.3
VL5	267.5	10.4	57.4	96.7	−85.3	182.0	0.089	34.5
