# Supplementary material for: Wing-kinematics measurement and aerodynamics in a small insect in hovering flight
Source: Sci Rep. 2016 May 11;6:25706. doi: 10.1038/srep25706 (PMC4863373; doi:10.1038/srep25706)
Supplement: Supplementary Information [file srep25706-s1.pdf]

Electronic Supplemental Material to "Wing kinematics measurement and aerodynamics in a small insect in hovering flight"

Xin Cheng* and Mao Sun

Institute of Fluid Mechanics, Beijing University of Aeronautics and Astronautics, China

(*Corresponding author: x.cheng@buaa.edu.cn)

S1 Error analysis of the measurement

We employed the same method of error analysis as that of Mou et al. [1]. Errors in the method came from several sources. One of them was the deformation of the flapping wings: the wings were not flat-plate wings as they were modeled in the method. Other error sources included errors due to camera model inaccuracy (pinhole model was used to characterize the camera), camera calibration errors, stereo rig calibration errors and discretization errors. It was shown that errors due to camera model inaccuracy, camera calibration and stereo rig calibration were small [1], and the primary errors of the method were errors due to wing deformation and discretization.

We estimated the wing deformation and discretization errors as a whole. We applied the method to a computer-generated virtual insect consisting of a rigid body and two deforming wings. The wings could rotate around their roots (being joints with three degrees of freedom), and had time-dependent twist and camber deformations. The body was unrestrained in space, having six degrees of freedom. The wing and body motions and the wing deformation of the virtual insect were as follows. The positional and elevation angles and the pitch angle at r_2 of the wing were set as the same as those of VL1. Based on the observations of the flight in the

flies, the wing was assumed to have a 8° twist and 4% camber during the translation phase of the downstroke or upstroke and the twist and camber increase to 14° and 7%, respectively, at stroke reversal. It was assumed that the center of mass of the body had a horizontal harmonic oscillation of a 0.05 mm amplitude and the pitch angle of the body had a harmonic oscillation of a 0.5° amplitude. Each wingbeat cycle contains 34 data points.

We first used the projection matrices [1] to project the virtual insect onto the image planes of the cameras (setting the resolution and pixel ratio the same as those of the real cameras) and obtained three nearly orthogonal projective images of the virtual insect (an example of the 3-D virtual insect and its images is given in Fig. S1). This step contains the discretization errors.

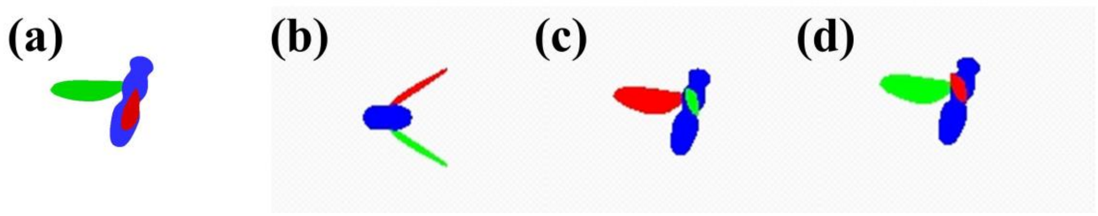

Figure S1. An example of the of the virtual insect (a) and its three projective images (b, c, and d).

Next, as was done in the real experiment, we represented the virtual insect by the body and wing models (body represented by a line segment and wing represented by its outline; each of the models was a set of points). We put the models of the body and the wings into the world coordinate system [1] and then changed the positions and orientations of the models until the best overlap between each model's projection and the corresponding image of the virtual insect was achieved in all three views. We thus obtained the measurements of body and wing kinematics of the virtual insect.

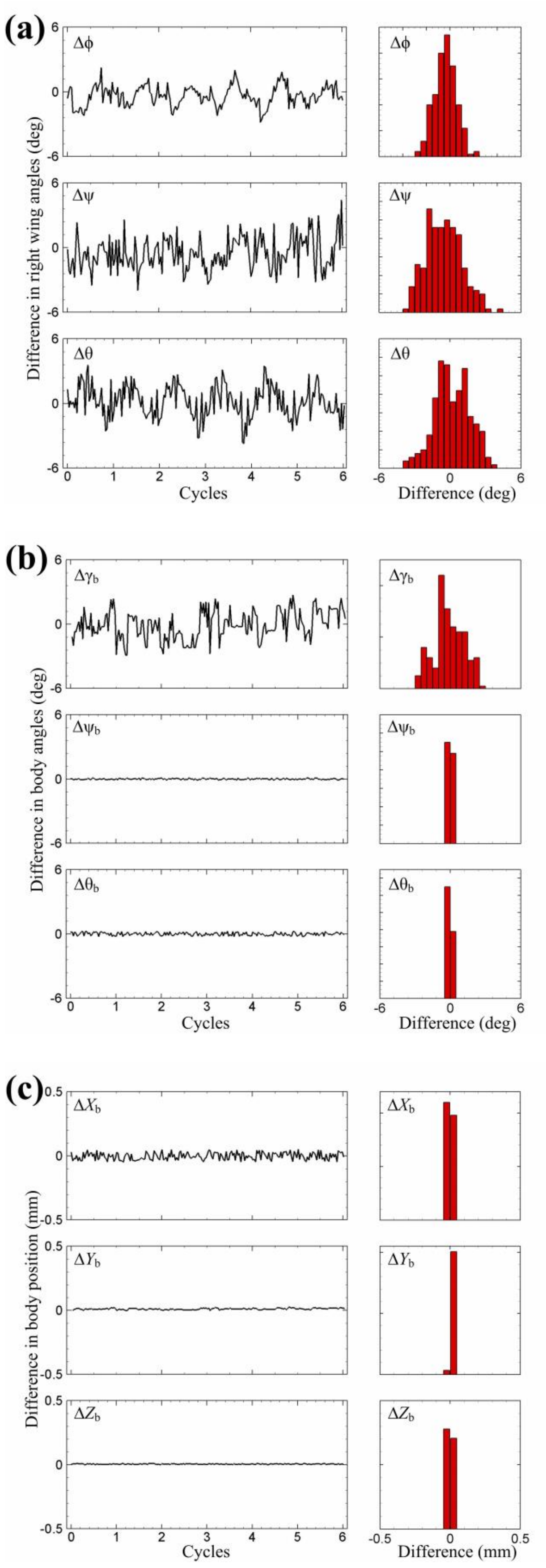

Figure S2. Differences between the measured and imposed wing and body kinematic parameters (measured value minus imposed value); ‘Δ’ denotes difference. (a) Wing angles: ϕ,

positional angle; θ, elevation angle; ψ pitch angle. (b) Body angles: ψ_b, yaw angle; θ_b, pitch angle; γ_b, roll angle. (c) Position of body center of mass: x_b, y_b, and z_b, horizontal, sidewise and vertical displacements of the body center of mass, respectively.

Comparison between the measured body and the imposed body and wing kinematics gave the errors. Six wingbeats were analyzed. Figure S2 shows the differences between the measured and imposed kinematic parameters and the corresponding histograms for the body and the right wing (results for the left wing are similar). As seen in Fig. S2a, for positional angle of the wing, errors are within 3° and the residuals are nearly centered around zero, indicating that there are only small systematic deviations; for the pitch angle and elevation angle of the wing, errors are a little larger, within 4°. For the body orientation (Fig. S2b), error in roll angle is within 3° and errors in yaw and pitch angles are approximately 1°. Errors in the body position (Fig. S2c) are typically less than 0.05mm or 3% of body length.

S2 Successive snapshots of a stroke cycle

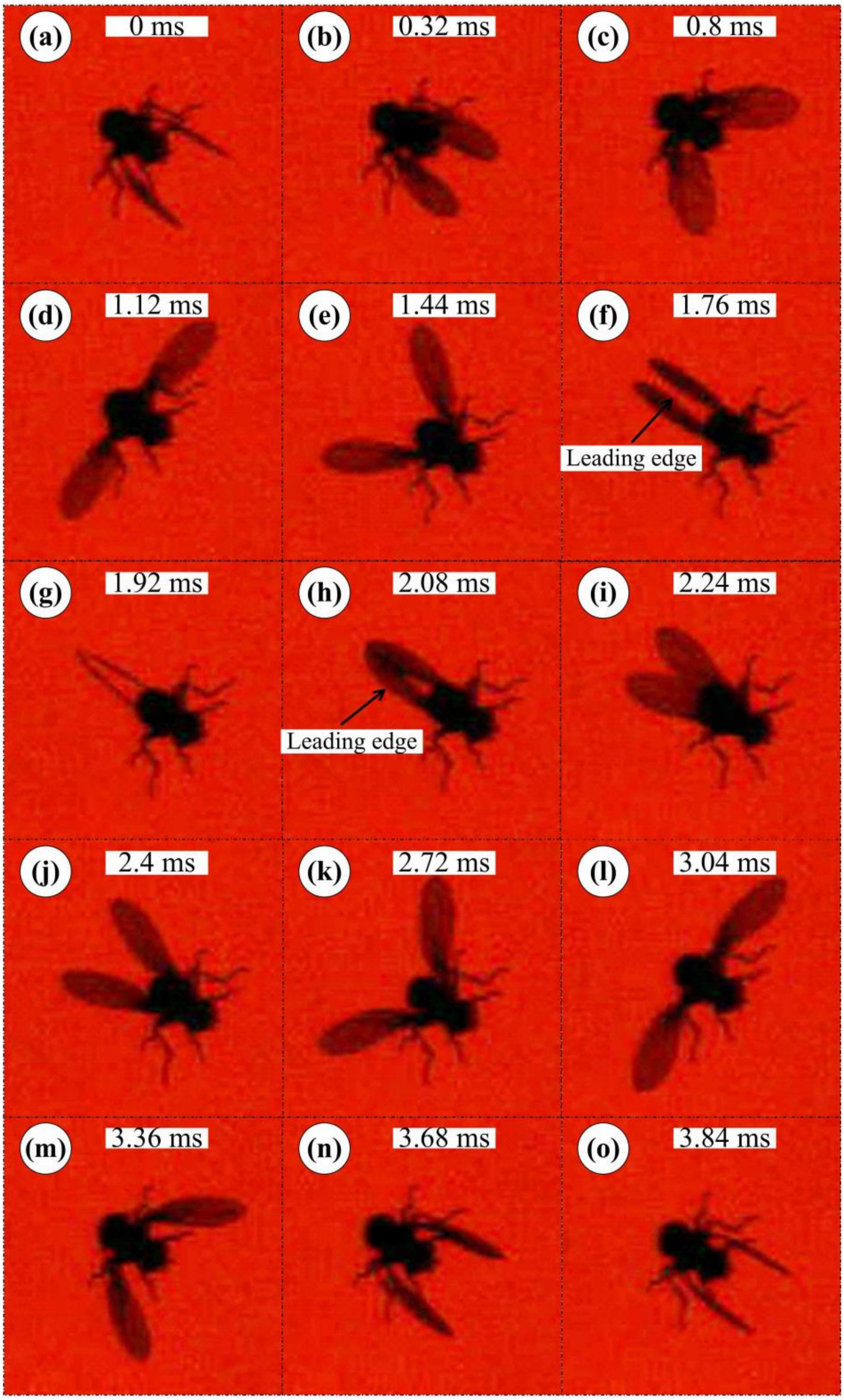

Figure S3. Successive snapshots of VL1 from one of the three cameras.

S3 Solution method of the Navier-Stokes equations

The computational method is the same as that used by Sun and Yu [2] and only

an outline of it is given here. The method is based on the artificial-compressibility algorithm, which was first developed by Rogers et al. [3] for single-grid, and then extended by Rogers and Pulliam [4] to overset grids. In the method, the time derivatives of the momentum equations were differenced using a second-order, three-point backward difference formula. To solve the time discretized momentum equations for a divergence free velocity at a new time level, a pseudo-time level is introduced into the equations and a pseudo-time derivative of pressure divided by an artificial compressibility constant was introduced into the continuity equation. The resulting system of equations were iterated in pseudo-time until the pseudo-time derivative of pressure approaches zero, thus, the divergence of the velocity at the new time level approached zero. The derivatives of the viscous fluxes in the momentum equation were approximated using second-order central differences. For the derivatives of convective fluxes, upwind differencing based on the flux-difference splitting technique was used. A third-order upwind differencing was used at the interior points and a second-order upwind differencing used at points next to boundaries. With overset grids, for each wing there was a body-fitted curvilinear grid, which extends a relatively short distance from the wing, and in addition, there was a background Cartesian grid, which extends to the far-field boundary of the domain. The solution method for single-grid was applied to each of these grids; data were interpolated from one grid to another at the inter-grid boundary points. Details of this algorithm can be found in Refs. [3,4]. For far field boundary conditions, at the inflow boundary, the velocity components were specified as free-stream conditions while pressure was extrapolated from the interior; at the outflow boundary, pressure was set equal to the free-stream static pressure and the velocity was extrapolated from the interior. On the wing surfaces, impermeable wall and no-slip boundary conditions

were applied, and the pressure on the boundary was obtained through the normal component of the momentum equation written in the moving coordinate system. The wing grids were generated using a Poisson solver which was based on the work of Hilgenstock [5]; they are of O-H type grids. The background Cartesian grid was generated algebraically.

Before proceeding to compute the flows, grid resolution tests were conducted to ensure that the flow calculation was grid independent. For a clear description of the time courses of the forces, we express the time during a cycle as a non-dimensional parameter, $\hat{t}$, such that $\hat{t}$=0 at the start of an upstroke, and $\hat{t}$=1 at the end of the subsequent downstroke. Three grid-systems were considered. For grid-system 1, the wing grid had dimensions $41\times61\times43$ around the wing, in the normal direction and in the spanwise direction, respectively (first layer grid thickness was 0.0015*c* where *c* is the mean chord length of wing), and the background grid had dimensions $81\times81\times81$ in the x_E, y_E and z_E directions, respectively. For grid-system 2, the corresponding grid dimensions were $61\times91\times65$ and $121\times121\times121$ (first layer grid thickness was 0.001*c*). For grid-system 3, the corresponding grid dimensions were $91\times135\times96$ and $181\times181\times181$ (first layer grid thickness was 0.00067*c*). In all the grid-systems, the outer boundary of the wing-grid was set at about 2.5*c* from the wing surface and that of the background-grid at 20*c* from the wings. For all the three grid-systems, grid points of the background grid concentrated in the near field of the wings where its grid density was approximately the same as that of the outer part of the wing-grid. As an example, portions of grid-system is shown in Figure S4. Figure S5 shows the computed lift (C_L) and drag (C_D) coefficients of a fly (VL1) in one cycle; results calculated with three grid-systems are plotted. Figure S6 shows the contours of the non-dimensional spanwise component of vorticity at 70% of the span

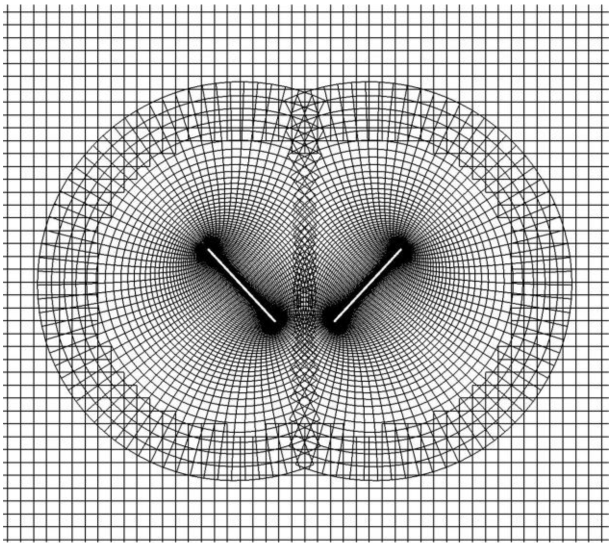

Figure S4. Portions of the moving overset grids.

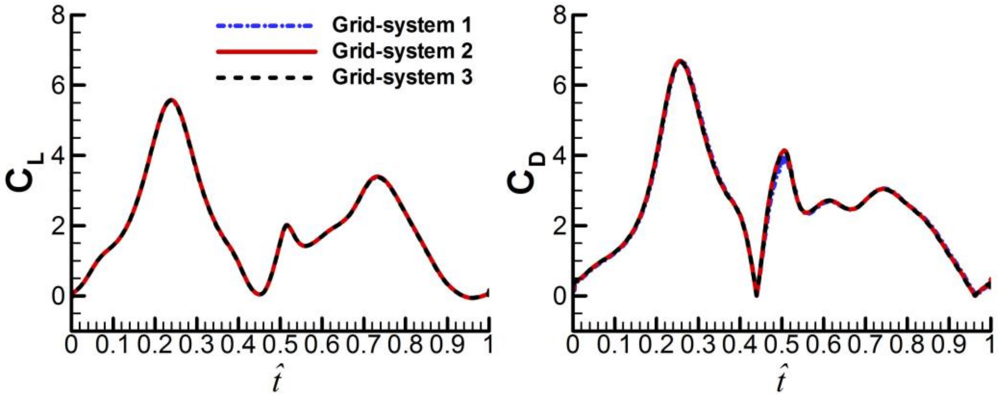

Figure S5. Time courses of the C_L and C_D in one cycle, calculated with three grid-systems.

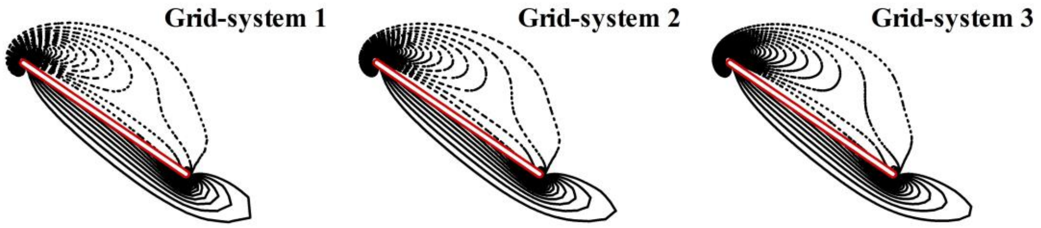

Figure S6. Contours of non-dimensional spanwise component of vorticity at 70% of the spanwise (the magnitude of the vorticity at the outer contour is 2 and the contour interval is 1).

at $\hat{t}$ =0.75. It is observed that the first grid refinement produces a little change in the vorticity plot, and the second grid refinement produces almost no change (Figure S2).

There is almost no difference between the force coefficients calculated by the three grid-systems (Figure S1). Calculations were also conducted using a larger computational domain. The domain was enlarged by adding more grid points to the outside of the background grid of grid-system 2. The calculated results showed that there was no need to put the outer boundary further than that of grid-system 2. The non-dimensional time step was 0.02 (non-dimensionalized by c/U where U is mean velocity of the wing at the radius of the second moment of wing area). The effect of time step value was studied and it was found that a numerical solution effectively independent of the time step was achieved if the time step value was ≤0.02. From the above discussion, it was concluded that grid-system 2 and time step was 0.02 were proper for the calculation.

S4 Calculations in which α is modified

For the flies, the stroke amplitude has already reached its limit (≈185°), and the angle of attack is around 40°, not small. It is of interest to see if the mean force coefficients could be further increased by increasing the angle of attack. We modified the angle of attack of VL1, as shown in Fig. S7 (α in the mid-portion of a down- or upstroke is increased by about 5°, 11° and 16°, respectively), and computed the flows. The computed results are given in Table S1 (the results are discussed in the main text).

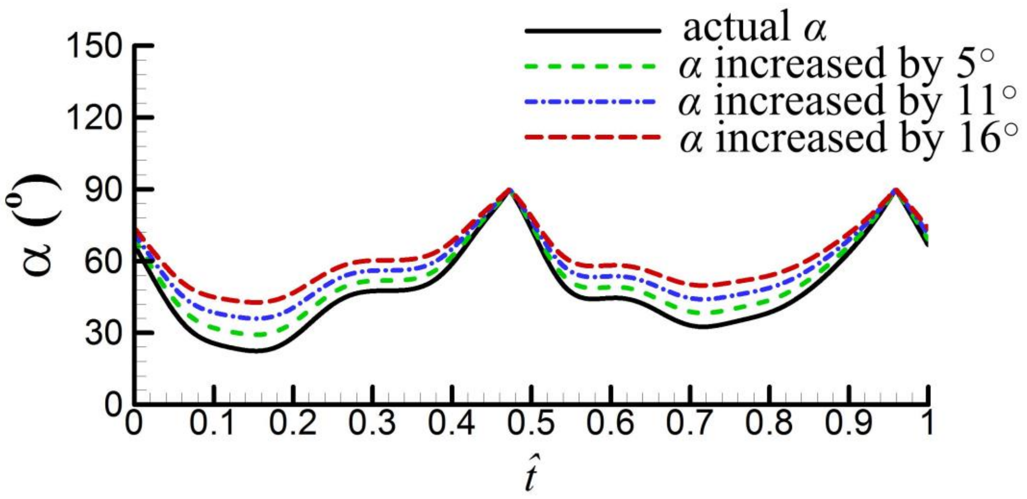

Figure S7. Actual and modified α of VL1in one cycle.

Table S1. Mean force coefficients of VL1 with actual and modified α.

	$\bar{C}_V$	$\bar{C}_D$
actual α	1.89	2.56
α increased 5 °	2.00 (6.0%)	2.91 (13.8%)
α increased 11 °	2.05 (8.5%)	3.29 (28.6%)
α increased 16 °	2.03 (7.5%)	3.68 (43.8%)

S5 Effect of elevation angle

In order to show the elevation-angle effect, θ of VL1 was set to zero and the flow computation was repeated. The computed C_L and C_D, compared with those with actual θ, are shown in Figure S8 (for convenience, the time variation of θ is also shown in the figure).

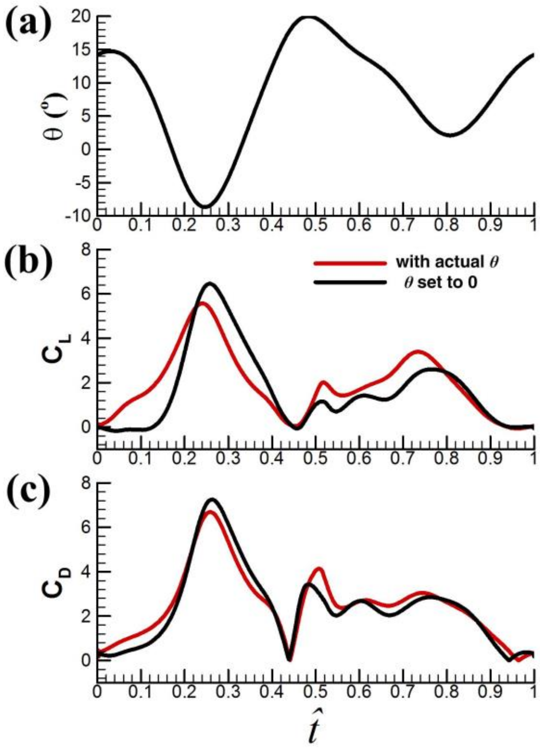

Figure S8. Time courses of C_L and C_D in one cycle for VL1, with and without elevation angles.

Let us first look at the upstroke ($\hat{t}$≈0–0.45). In the first part of the half-stroke ($\hat{t}$≈0–0.24), C_L and C_D are increased by the elevation angle; in the next part of the half-stroke ($\hat{t}$≈0.24–0.45), they are decreased. A possible explanation for this is as following. In the first part of the half-stroke ($\hat{t}$≈0–0.24), θ decreases (Figure S8a) and the translating wing has an additional downward velocity. This would increase the effective angle of attack of the wing, resulting the increase in the aerodynamic forces. In the next part of half-stroke ($\hat{t}$≈0.24–0.45), θ increases (Figure S8a) and the translating wing has an additional upward velocity, which would reduce its effective angle of attack, resulting the decrease of the aerodynamic forces. The results in the downstroke (Figure S8, $\hat{t}$≈0.45–1.0) can be similarly explained.

From Figure S8, it can also be observed that although the elevation anlge produces the above effects, the general time-variation of the force coefficients are qualitatively similar between the cases with and without deviation angle.

Supplementary Movie 1. VL1 hovering. The left, middle and right parts of the movie show the flight captured by the top-view camera, side-view camera and back-view camera, respectively. Playback speed is 10fps, approximately 0.16% of the actual speed of the movie (The movie was compressed with TMPGEnc 3.0 XPress).

Supplementary References

1. Mou, X. L., Liu, Y. P., Sun, M. Wing motion measurement and aerodynamics of hovering true hoverflies. *J. Exp. Biol.* **214**, 2832-2844 (2011).
2. Sun, M., Yu, X. Aerodynamic force generation in hovering flight in a tiny insect. *AIAA J.* **44**, 1532-1540 (2006).
3. Rogers, S. E., Kwak, D., Kiris, **C.** Steady and unsteady solutions of the incompressible Navier-Stokes equations. *AIAA J.* **29**, 603-610 (1991).
4. Rogers, S. E., Pulliam, T. H. Accuracy enhancements for overset grids using a defect correction approach *AIAA, Aerospace Sciences Meeting and Exhibit, 32 nd.* 94-0523 (1994).
5. Hilgenstock, A. A fast method for the elliptic generation of three dimensional grids with full boundary control. in *Num. Grid Generation in CFM'88* (eds Sengupta, S. *et al.*). 137-146 (Ld, 1988).
